# Formulation of nanoemulsion parijoto fruit extract (*Medinilla Speciosa*) with variation of tweens stabilizers

**DOI:** 10.3389/fnut.2024.1398809

**Published:** 2024-07-08

**Authors:** Victoria Kristina Ananingsih, Alberta Rika Pratiwi, Bernadeta Soedarini, Yohanes Alan Sarsita Putra

**Affiliations:** Department of Food Technology, Soegijapranata Catholic University, Semarang, Indonesia

**Keywords:** nanoemulsion, stabilizers, tween, parijoto, RSM

## Abstract

Nanotechnology has substantial potential for development due to its ability to modify surface characteristics and particle size, facilitating enhanced absorption of functional food compounds and controlled release of active substances to mitigate adverse effects. Nanoemulsion, a stable colloidal system formed by blending oil, emulsifier, and water, was identified as nanotechnology with promising applications. However, investigations into the impact of surfactants on characteristic nanoemulsions need to be more varied. This research gap necessitated further exploration in the advancement of nanotechnology-based foods. The parijoto fruit (*Medinilla speciosa*), an indigenous plant species in Indonesia, has yet to undergo extensive scrutiny for its potential use as a functional and nutraceutical food. Anthocyanins, a principal compound in the parijoto fruit, had exhibited efficacy in reducing the risk of cardiovascular disease diabetes, demonstrating anti-inflammatory and antioxidant properties. This study aimed to investigate the characteristics of nanoemulsion formulations derived from parijoto fruit extract and to evaluate an optimum condition with various tween surfactants. The findings from this investigation could furnish valuable insights for the further advancement of anthocyanin nanoemulsions from parijoto fruit extract. The results comprised the characterization of nanoemulsion particle size, polydispersity index, ζ-potential, conductivity, pH, and viscosity. Through mathematical modeling and statistical methods, RSM optimizes nanoemulsion by examining the relationships and interactions between independent and response variables. Furthermore, the characterization of nanoemulsion encompassed ζ-potential, polydispersity, particle size, conductivity, pH, and viscosity. Elevated surfactant concentrations resulted in diminished particle sizes and more uniform size distribution, albeit reaching a plateau where surfactant aggregation and micelle formation ensued. Increased concentrations of surfactant type, concentration, and parijoto extract impacted the physical characteristics of nanoparticle size and polydispersity. The optimal process conditions for nanoemulsion consisting of the type of Tween used are Tween 80, Tween concentration of 12%, and parijoto fruit extract concentration of 7.5%, yielding a desirability value of 0.74, categorizing it as moderate.

## 1 Introduction

Nanotechnology underwent progressive evolution, characterized by measurements on the nanometer scale, approximately 10^−9^ meters (1). Acknowledgment from the World Health Organization (WHO) and the Food and Agriculture Organization (FAO) (2) underscored nanotechnology’s significant potential in enhancing food products, attributed to its capacity to modify surface characteristics and particle size. Such modifications facilitate targeted delivery of food compounds to specific organs and the controlled release of active compounds to mitigate adverse effects. The attributes of nanoscale food materials are pivotal in propelling diverse industries, including food, pharmaceuticals, and extensive nutraceutical applications (3). Due to their substantial surface area-to-volume ratio, nanoemulsions exhibit enhanced stability against gravitational separation and aggregation, owing to their distinct physicochemical and biological characteristics compared to conventional emulsions. The droplets or globules inherent in nanoemulsions mitigate gravitational forces and Brownian motion, thereby averting creaming or sedimentation during storage. Nanoemulsions denote a nanotechnological rendition of a stable colloidal system, achieving kinetic stability through the amalgamation of oil, emulsifier, and water (4). Chang et al. (5) conducted research utilizing surfactants as stabilizers in synthesizing nanoemulsions, showcasing the stability of nanoemulsion particle size in curcumin extract. Surfactants can diminish interfacial tension and form a substantially influential steric elastic film on the emulsion results (6).

Renowned for its tropical climate and vast biodiversity, Indonesia harbors at least 30,000 plant species, with 7,000 being herbal plants with documented health benefits (7, 8). Parijoto (*Medinilla speciosa*), an endemic plant species in Indonesia, remains relatively understudied for its scientific potential in pharmacy, functional foods, and nutraceuticals. Analysis has confirmed that the parijoto fruit comprises phytochemical components such as anthocyanins, flavonoids, saponins, tannins, alkaloids, cardenolides, and glycosides. Anthocyanins, a predominant compound in parijoto fruit, demonstrate efficacy in reducing the risk of cardiovascular diseases, diabetes, and inflammation while possessing notable anti-inflammatory and antioxidant properties. Extraction techniques yield varying anthocyanin contents, with the peel extract and whole fruit extract registering 208.75 and 173.7 mg/L, respectively (9). Various factors influence anthocyanins’ stability, including chemical structure, concentration, solvent, pH, storage temperature, light, oxygen, metal ions, proteins, and flavonoids. Weak stability under high pH, high temperature, and light exposure has been observed, with lower pH contributing to enhanced stability (10, 11). Heating at elevated temperatures accelerates anthocyanin degradation (12).

Response Surface Methodology (RSM) has emerged as a prominent multivariate statistical technique for optimizing various processes in recent years. Initially introduced by Box and colleagues in the 1950s, RSM facilitates examining the relationship and interactions among independent and response variables through mathematical modeling and statistical methods (13). RSM has successfully enhanced and optimized therapeutic extract and drug nanoemulsion (14). In this study, Central Composite Design (CCD) Response Surface Methodology (RSM) was employed to optimize the quality parameters of the nanoemulsion.

Appropriate nano-encapsulation techniques, such as nanoemulsion, have shown the potential to enhance the stability, bioavailability, and solubility of lipophilic bioactive compounds while also preventing hydrolysis and oxidation (15). The encapsulation process aimed to protect the active substance from oxidation by air and light, thereby increasing the shelf life of the product (16). Nanoemulsions are widely utilized nanoformulations in food-related industries through active or passive targeting mechanisms. Gunasekaran et al. (17) introduced nanotechnology as an effective tool for enhancing the bioavailability and bioactivity of phytomedicine. Nanoemulsion has emerged as a novel technology, providing opportunities to address challenges associated with delivering micronutrients in functional food. Shin et al. (18) explored recent advancements in nanoformulation of lipophilic functional foods. Moreover, nanotechnology-based strategies have been explored to associate complex matrices derived from plant extracts, offering promising prospects for developing novel therapeutic formulations (19). Synthesis of nanoemulsion using mangosteen peel extract rich in anthocyanins as the main ingredient of the formulation can increase the dominant penetration of α-mangostin through the stratum corneum (20). Catechin nanoemulsion showed a remarkable improvement in stability and bioavailability in simulated gastrointestinal conditions (21). Mulia et al. (22) showed the optimum results using a high-speed homogenization and Tween surfactant to prepare nanoemulsions with nanoemulsion. Research conducted by Chang et al. (5) used Tween as the surfactant in the stable nanoemulsion synthesis loaded curcumin extract. This highlights the opportunity to develop nanoemulsion formulations for anthocyanins found in parijoto fruit. So far, research on nanoemulsion formulation in parijoto fruit involving various concentrations and stabilizers still needs to be conducted. This current study investigates the characteristics of nanoemulsion formulations derived from parijoto fruit extract and evaluates an optimum condition with various tween surfactants.

## 2 Materials and method

### 2.1 Materials

Grinder (Binder), Erlenmeyer (Pyrex), beaker glass (Pyrex), volume pipette, test tube (Pyrex), test tube rack, funnel (Pyrex), measuring flask (Pyrex), vacuum n filter 0.22 nm (Sartorius Stedim 11,694–2–50-06), vial, micropipette (Socorex), blue tip (Biologix 1 nmL pipette tips), hotplate (Cimarec et al. SP142025Q), vortex (Thermolyne et al.), Ultrasonic Cleaner (Biobase UC-10SD) modified, UV–VIS spectrophotometer (Shimadzu, UV-1280), aluminum foil, filter paper, 0.22 μm filter membrane (Wattman), Cabinet dryer (HetoPowerDry LL1500), rotary evaporator (Biobase RE-2000E), syringe, analytical balance. Fresh parijoto, ethanol pro analysis (Merck, Germany), methanol pro analysis (Merck, Germany), distilled water, F. ciocalteu 10% (Merck, Germany), Na2CO3 7.5% (Merck, Germany), DPPH solution (Merck, Germany), Quarcetin (Merck, Germany), AlCl3 (Merck, Germany), ammonium acetate 1 M(Merck, Germany), acetone (Merck, Germany), acetonitrile (Merck, Germany), standard cyanide (Zigma), delphinidin glu standard (Zigma), Tween 20 (Merck, Germany), Tween 60 (Merck, Germany), and Tween 80 (Merck, Germany).

### 2.2 Preparation of dry samples of parijoto fruit extract

Samples used in this study are fruits from the Parijoto plant (Medinilla speciosa) cultivated and harvested on the slopes of Mount Muria, Kudus. The fruits used are ripe fruits harvested when the Parijoto plant reaches full maturity, typically around 90–100 days after flowering. Parijoto, which had been cleaned and sorted, was weighed 200 grams for each treatment. The fruit that has been weighed is then steam-blanched for 3 min. Prepare a citric acid solution with a concentration of 1% for pre-treatment of fruit before drying. After that, soak the parijoto fruit in the citric acid solution for 5 min and drain. The Cabinet Dryer is cleaned before use to maintain hygiene and avoid cross-contamination. The drying temperature used was 70°C for 6 h. The dried Parijoto fruit is then ground into powder using a herbal grinder for 2 min. After that, the sample will be extracted for further testing. The dried Parijoto will be chemically analyzed using UV–Vis spectroscopy.

### 2.3 Making parijoto extract using ultrasonic assisted extraction

Five grams of dry sample powder and 50 mL of 99.5% ethanol were mixed thoroughly for homogeneity in four 250 mL centrifuge bottles. Then, all vials were sonicated using a Bio-Based Ultrasonic Waterbath with a 40 KHz frequency and 100 W power for 30 min. Subsequently, the samples were subjected to shaking for 1 h. The centrifugation step was performed at 4,000 rpm at 4°C for 10 min (Ohaus, United States). The supernatant was then carefully collected, and the remaining solution was evaporated to dryness under vacuum conditions. The residue was dissolved in 99.5% ethanol and diluted to 20 mL. After filtering through a 0.22 μm membrane filter, parijoto fruit extract was obtained and stored at −20°C for UV–Vis analysis.

### 2.4 Preparation of anthocyanin nanoemulsion from parijoto extract

Approximately 3 mL of anthocyanin nanoemulsion with concentrations of 2 mg/mL, 4 mg/mL, and 6 mg/mL, respectively, were prepared by collecting a portion of parijoto extract, and the solvent was removed with nitrogen. The solvent removal process during anthocyanin extraction can be monitored using a combination of visual inspection and periodic weight measurements. Visual inspection involves observing the extract as the solvent evaporates, noting its increasing concentration, evidenced by a thicker and more viscous appearance. Periodic weighing of the container or flask containing the extract allows for weight loss tracking as the solvent evaporates. Once the weight stabilizes or reaches a predetermined target, the desired solvent removal rate has been attained, ensuring the production of a concentrated anthocyanin extract suitable for further analysis. Anthocyanin nanoemulsion was prepared using a combination of surfactants that have low, medium, and high hydrophile lipophile balance (HLB), namely Twen 20, Tween 60, and Tween 80. Then, surfactant (0.24 g) was added, and the mixture was homogenized entirely. This was followed by adding (2.76 g) deionized water and mixing again for complete dispersion of surfactant in water. The solution was then sonicated in a sonicator with a temperature of 35°C, frequency of 45 Hz, and 100% power for 60 min. To produce a good nanoemulsion, homogenization was carried out using high shear homogenization at 15,000 rpm with a temperature of 4\u00B0C for 15 min.

### 2.5 Characterization of particle size and polydispersity index of nanoemulsion parijoto fruit extract

The particle size analysis tool used in this study was the Zetasizer (Zetasizer Pro; Malvern Instruments, Ltd., Malvern.), which operates based on the general principle of dynamic light scattering (DLS). This tool has a detector placed at an angle of 173 ° from the transmitted light beam and detects size using a patented technology known as noninvasive backscattering. This technique is used for various purposes. One is to reduce the effect known as multiple scattering, making it easier to measure samples with high concentrations. Modifying McClements (2016), the particle size distribution and average particle size of nanoemulsions were determined by dynamic light scattering (DLS) at a wavelength of 633 nm and a temperature of 25°C.

### 2.6 Characterization of Ζ-potential nanoemulsion parijoto fruit extract

The ζ-potential of Parijoto Fruit Extract Nanoemulsion was evaluated using ζ-potential analysis (Zetasizer Pro; Malvern Instruments, Ltd., Malvern) following the method described by Khalid et al. (2017). The ζ-potential of the samples was evaluated automatically using 10 to 100 analytical runs after equilibration for 120 s at 25°C. The ζ-potential of the particles was measured by phase-analysis light scattering (PLS) using a Zeta dip cell.

### 2.7 Characterization of the conductivity of nanoemulsion parijoto fruit extract

The conductivity of nanoemulsion particles was measured by phase-analysis light scattering (PLS) using a Zeta dip cell with a cuvet electrode. Samples were evaluated automatically using 10 to 100 analytical runs after equilibration for 120 s at 25°C. The detector is placed at an angle of 173° from the transmitted light beam.

### 2.8 pH measurement of nanoemulsion parijoto fruit extract

The pH was determined using a Schott pH meter at room temperature (27 ± 2°C), calibrated with a standard buffer of pH 7. The pH analysis of the Parijoto fruit extract nanoemulsion sample was carried out using a pH meter with a particular electrode. First, the pH meter is set and calibrated with a standard buffer solution at a known pH, generally at pH 4.0, 7.0, and 10.0. Samples were diluted with 10 mM phosphate buffer pH seven before analysis to avoid multiple scattering effects during testing. The pH meter electrode is then carefully inserted into the sample to ensure good contact. Once the electrode is stable, a pH reading is taken and recorded. This step is repeated as necessary to obtain consistent results. This pH analysis provides essential information regarding the acidity or alkalinity level of nanoemulsion and nanocitosan Parijoto fruit extract, which can affect the stability and quality of products using the nanoemulsion.

### 2.9 Viscosity measurement of nanoemulsion parijoto fruit extract

Viscosity measurements are carried out using a viscometer brookfield. 14 mL of sample was put into the cup and attached to the solvent trap provided. The viscometer was set at 200 rpm, three rotations, for 30 s. The measurement process begins by activating the viscometer, and this tool automatically measures the time required for a liquid to flow through the viscometer tube at a specific temperature and rpm. This time, a predetermined formula converts the reading into a viscosity value. Repeated measurements can be made to ensure consistent results.

### 2.10 Statistical analysis uses response surface methodology

In this study, primary data in 3 repetitions of extraction and three repetitions of testing were averaged and given a standard deviation value for each treatment combination using Statistica 12.5 by StatSoft. The data is then entered into a statistical application, arranged in a combination of factorial points, axial points, and central points with three repetitions. After that, the data was analyzed, and several test stages were carried out. The basis for testing is studentification from primary data. Studentification means that the scale of the variable is adjusted by dividing it by the estimated population standard deviation. Variability in sample standard deviation values contributes to additional uncertainty in the calculated value. This will cause problems in finding the probability distribution of each statistic studied.

#### 2.10.1 Effect summary

This test can summarize the effects of the combination of treatments used. The Longworth value in the results of this test is defined as-log (*p*-value) and is a transformation of the p-value based on the Pearson Chi-Squared test. The Pearson Chi-Squared test evaluates the possibility of the split being caused by chance. The higher the Pearson Chi-Squared value, the higher the probability of the split being caused by dependency. Generally, if the worth is greater than 2, the statistical model considers the variable necessary.

#### 2.10.2 Lack of fit

Model suitability testing (lack of fit) is carried out to review whether the model equation is acceptable or not in predicting responses. In the lack of fit test, the following hypothesis is used:

H0 = no lack of fit (suitable model).

H1 = there is a lack of fit (the model is not suitable).

The hypothesis is concluded by comparing the calculated *F* value with the F table. The calculated F is obtained from the statistical test results and displayed in the ANOVA table. The F table value is obtained from the F Distribution Table. The criteria for the lack of fit test are:

F count < F table, then H0 is accepted. F count > F table, then H0 is rejected.

Another parameter that can prove the suitability of the model obtained is by comparing the *p*-value with the α value. If the *p*-value of lack of fit is smaller than the α value, then there is a significant lack of fit, so the model obtained is inappropriate.

#### 2.10.3 Summary of fit

The R square and Root Mean square error values are obtained in this test. Measures the difference in values from a model’s predictions as estimates of the observed values. R square is also known as the coefficient of determination, which explains how far independent data can explain dependent data. R square has a value between 0 and 1 with the condition that the closer it is to one, the better it is. If the r square is 0.6, the independent variable can explain 60% of the distribution of the dependent variable. The independent variable cannot explain the remaining 40% or can be explained by variables outside the independent variable (error component).

#### 2.10.4 Parameter estimates

The parameter estimates are the coefficients of the linear predictor. This value represents the change in response if you have a certain level of a categorical predictor or a change of 1 unit for a continuous predictor, which means the same thing as in a multiple regression analysis with continuous response.

#### 2.10.5 Analysis of variance

The ANOVA test (Analysis of Variance) has the following test criteria:

H0 is accepted if F count < F table, which means the model cannot be accepted statistically because no independent variables directly influence the response.

H1 is accepted if F count > F table, which means the model is statistically acceptable and at least one independent variable has a real influence on the response.

#### 2.10.6 Fitted surfaces

The depiction of the fitted surface is carried out using the Central Composite Design model. The experimental design is factorial, specifically Central Composite Design (CCD). CCD was chosen over Box–Behnken Design because CCD provides more design points in terms of axial points. Additionally, CCDs can run experiments at extreme values, providing better quadratic equations for analysis. CCD contains a factorial or fractional factorial design with a central point augmented by a group of ‘axial points’ that allow estimation of curvature. If the distance from the center of the design space to the factorial point is ±1 unit for each factor, the distance from the center of the design space to the axial point is | α | > 1. The exact value of α depends on the properties desired for the design and the number of factors involved. The CCD has twice as many star points due to a factor in the design.

## 3 Result and discussion

### 3.1 Phytochemical profiles of dried parijoto fruit

Drying Parijoto Fruit is carried out using a cabinet dryer at a temperature 70°C for 6 h. The results of drying parijoto fruit were obtained through the preparation process; the antioxidant and anthocyanin activity profiles were expressed, respectively, in units of % inhibition and ppm. The total anthocyanin content in the dry samples and extracts was 538.47 ± ppm. The dried Parijoto exhibited significant antioxidant activity, with a % inhibition value of 79.14 ± 34.82. This indicates a substantial capacity to neutralize free radicals in various chronic diseases and aging processes. The high antioxidant activity suggests that the drying process did not significantly diminish the antioxidant potential of Parijoto. The total anthocyanin content of the dried Parijoto was 538.47 ± 4.67 ppm. Anthocyanins are a group of pigmented compounds known for their antioxidant properties and potential health benefits. The retention of anthocyanins after drying indicates that cabinet drying effectively preserved these bioactive compounds in the dried Parijoto.

The parijoto fruit extract was obtained through an extraction process using the Ultra-assisted extraction method. The Ultra-assisted extraction method involves the utilization of a modified ultrasonic water bath for the extraction of parijoto fruit. This method harnesses ultrasonic energy to enhance the extraction process by facilitating cell wall breakdown and increasing target compounds’ solubility. During extraction, the parijoto fruit is immersed in a solvent within the ultrasonic waterbath, where ultrasonic waves are applied to the sample. This results in intensified agitation and cavitation within the solvent, leading to improved extraction efficiency and higher yields of bioactive compounds from the fruit. The characterization of Parijoto extract as a filler in nanoemulsion involved various analyses to assess its antioxidant properties and phytochemical composition. The extraction method was ultra-assisted extraction, known for its efficiency in extracting bioactive compounds from plant materials. The antioxidant activity of the Parijoto extract was evaluated, yielding a % inhibition value of 50.776 ± 6.18. This indicates a significant antioxidant capacity, crucial for combating oxidative stress and preventing cellular damage caused by free radicals.

Furthermore, the total anthocyanin content of the extract was determined to be 94.43 ± 4.14 ppm. Anthocyanins are well-known antioxidants in many fruits and vegetables. They are known for their potential health benefits, including anti-inflammatory and anti-cancer properties. The flavonoid content of the Parijoto extract was measured to be 126.85 ± 1.15 g/L. Flavonoids are a class of polyphenolic compounds known for their antioxidant and anti-inflammatory effects. Additionally, the phenolic content of the extract was quantified as 8.43 ± 0.70 GAE/g. Phenolic compounds are another group of bioactive compounds found in plants, known for their antioxidant and anti-inflammatory activities and their potential role in reducing the risk of chronic diseases.

### 3.2 Fitting model for RSM (response et al.) in parijoto fruit extract nanoemulsion

Data recorded for each run included nanoemulsion particle size, polydispersity index, ζ-potential, conductivity, pH, and viscosity. Each variable was measured with three repetitions and the measurements three times to get consistent results. This data will be used to analyze the influence of various factors on the characteristics of nanoemulsions using the Response Surface Methodology method, which can be seen in the table.

Table 1 shows that the particle size range of the nanoemulsion is between 14,603 ± 16.73 nm and 118,053 ± 4.5825 nm. The largest and smallest nanoparticle sizes found are 126.47 nm and 13.72 nm, respectively, with most nanoparticle sizes falling within the 50–100 nm range. Similar results were confirmed by Noor El-Din et al. (23), who reported nanoemulsion sizes ranging from 31.58 to 220.5 nm. Studies conducted by Delmas et al., Liu et al., and Mei et al. using ultrasonication and high emulsification methods also confirmed comparable results of 45–170 nm, 222.4–166.4 nm, and 170–280 nm, respectively (24–26). Conversely, Peng et al. (27) reported a nanoparticle size range of 21–684 nm. Ζ-potential reflects the surface charge of particles and affects colloidal stability. High ζ-potential can prevent particle aggregation due to electrostatic repulsion. The research includes the evaluation and characterization of ζ-potential under various treatments. The study obtained ζ-potential results for nanoemulsion ranging from −22.197 ± 0.738 mV to −28.207 ± 1.598 mV, respectively. Similar results were confirmed by obtaining results of +21.5 mV. Particles with high ZP values, between 20 and 40 mV, provide system stability and are less likely to aggregate or increase particle size. However, it should be noted that ZP values are not an absolute measure of nanoparticle stability. Furthermore, emulsions with ZP variations >10 mV are suggested to have better stability (28). The ideal potential range for nanoparticle stability is (−30 to 20 mV or + 20 to +30 mV) (25). The produced values tend to be harmful due to the influence of acetic acid, resulting in a negative charge. This charge causes electrostatic repulsion forces between formed nanoparticles to prevent aggregation into larger sizes. Higher ζ-potential values increase nanoparticle stability due to higher electrostatic repulsion forces between nanoparticles.

**Table 1:** Design of experiment RSM particle size, poly dispersity index, Ζ-potential, conductivity, pH, viscosity in nanoemulsion.

Dependent variables				Independent variables																	
No. run test	Types of lyphophilic tweens	Tween concentration (%)	Parijoto fruit extract concentration (%)	Nanoparticle size (nm)			Ζ-potential			Conductivity			Poly dispersity index			pH			Viscosity (Cp)		
	X_1_	X_2_	X_3_	Y_1_			Y_2_			Y_3_			Y_4_			Y_5_			Y_6_		
1	20	8	3	15.533	±	8.329	−24.14	±	1.574	0.071	±	0.005	0.43	±	0.05	6.747	±	0.035	0.071	±	0.005
2	20	8	3	19.2	±	9.337	−25.473	±	2.687	0.067	±	0.018	0.54	±	0.06	6.870	±	0.020	0.067	±	0.018
3	20	8	3	14.603	±	16.738	−22.230	±	1.421	0.066	±	0.001	0.57	±	0.01	6.760	±	0.030	0.066	±	0.001
4	20	8	6	22.667	±	13.903	−25.913	±	3.130	0.067	±	0.001	0.58	±	0.05	6.890	±	0.010	0.067	±	0.001
5	20	8	6	39.857	±	6.983	−25.157	±	2.342	0.069	±	0.016	0.48	±	0.07	6.753	±	0.025	0.069	±	0.016
6	20	8	6	41.593	±	8.759	−22.197	±	0.738	0.074	±	0.006	0.65	±	0.07	6.853	±	0.032	0.074	±	0.006
7	20	8	9	62.547	±	8.003	−24.643	±	3.787	0.074	±	0.016	0.52	±	0.13	6.897	±	0.006	0.074	±	0.016
8	20	8	9	64.343	±	3.707	−24.477	±	3.579	0.074	±	0.017	0.50	±	0.03	6.810	±	0.026	0.074	±	0.017
9	20	8	9	68.567	±	13.012	−24.037	±	0.699	0.064	±	0.013	0.40	±	0.04	6.817	±	0.025	0.064	±	0.013
10	20	10	3	58.677	±	16.690	−24.607	±	1.206	0.085	±	0.013	0.61	±	0.01	6.750	±	0.026	0.085	±	0.013
11	20	10	3	58.567	±	9.007	−24.437	±	1.253	0.090	±	0.010	0.56	±	0.06	6.863	±	0.021	0.090	±	0.010
12	20	10	3	56.237	±	3.897	−24.273	±	1.973	0.069	±	0.001	0.61	±	0.02	6.830	±	0.010	0.069	±	0.001
13	20	10	6	62.94	±	4.251	−25.570	±	1.207	0.068	±	0.029	0.59	±	0.04	6.897	±	0.006	0.068	±	0.029
14	20	10	6	64.687	±	9.472	−25.797	±	1.445	0.075	±	0.017	0.52	±	0.09	6.867	±	0.015	0.075	±	0.017
15	20	10	6	64.827	±	9.866	−25.150	±	2.092	0.087	±	0.010	0.59	±	0.04	6.780	±	0.010	0.087	±	0.010
16	20	10	9	72.297	±	12.209	−26.127	±	1.756	0.071	±	0.032	0.55	±	0.11	6.843	±	0.021	0.071	±	0.032
17	20	10	9	76.503	±	1.102	−26.807	±	0.081	0.085	±	0.024	0.53	±	0.03	6.880	±	0.020	0.085	±	0.024
18	20	10	9	77.593	±	19.672	−26.203	±	1.550	0.076	±	0.023	0.60	±	0.01	6.760	±	0.030	0.076	±	0.023
19	20	12	3	83.69	±	11.625	−26.007	±	1.514	0.082	±	0.015	0.87	±	0.10	6.890	±	0.010	0.082	±	0.015
20	20	12	3	85.4	±	14.626	−26.003	±	1.065	0.080	±	0.017	0.68	±	0.37	6.773	±	0.012	0.080	±	0.017
21	20	12	3	86.967	±	7.607	−26.053	±	3.298	0.085	±	0.016	0.64	±	0.33	6.777	±	0.032	0.085	±	0.016
22	20	12	6	97.49	±	2.250	−27.477	±	0.961	0.081	±	0.016	0.70	±	0.14	6.810	±	0.010	0.081	±	0.016
23	20	12	6	110.86	±	19.92	−27.077	±	1.709	0.081	±	0.017	0.57	±	0.27	6.870	±	0.020	0.081	±	0.017
24	20	12	6	116.23	±	7.094	−27.220	±	0.350	0.077	±	0.011	0.72	±	0.20	6.890	±	0.017	0.077	±	0.011
25	20	12	9	117.667	±	17.129	−28.207	±	1.598	0.078	±	0.010	0.64	±	0.29	6.873	±	0.021	0.078	±	0.010
26	20	12	9	118.33	±	4.583	−28.023	±	0.179	0.073	±	0.014	0.83	±	0.19	6.887	±	0.006	0.073	±	0.014
27	20	12	9	124.187	±	4.582	−28.070	±	0.634	0.089	±	0.010	0.73	±	0.21	6.887	±	0.015	0.089	±	0.010

Conductivity provides information about the ability of nanoemulsions to conduct electricity. Changes in conductivity can occur with changes in surface particle charge. Table 1 shows that the nanoemulsion conductivity of Parijoto fruit extract ranges from 0.03458 to 0.09987 mS/cm. Good nanoemulsion conductivity measurements have higher electrical conductivity values (10–100 μS/cm) (29). Electrical conductivity values tend to decrease with decreasing water content in the emulsion. O/W type (Oil-in-Water) nanoemulsions have higher conductivity than W/O type (Water-in-Oil) nanoemulsions. This is because the more extensive water phase provides more pathways for ion conduction.

The type and concentration of surfactant in nanoemulsion can influence conductivity. Surfactants can provide ionic charge or facilitate ion conduction in the system. Viscosity is an essential parameter in evaluating the flow properties of nanoemulsion. Viscosity is one of the parameters used to determine the stability of polymers in a solution because it undergoes reduction during polymer storage due to polymer degradation (30). In this study, as shown in Table 1, the viscosity of nanoparticles ranges from 3,810 cP to 4,433 cP. Alemu et al. (31) stated that viscosity can depend on particle size and storage time. Appropriate viscosity can affect the applicability and spread of the system. The viscosity of a preparation is related to the consistency and spreadability of the preparation, which will affect ease of use. Viscosity values are influenced by several factors, such as temperature, pH, manufacturing conditions, and the quality and concentration of raw materials. The results of viscosity tests are shown in centipoise (cP). The higher the viscosity value of a preparation, the better the stability of the product, but the preparation will be difficult to apply.

This ANOVA table is essential to evaluate the statistical significance of each model component and determine whether the quadratic model used is good enough to explain the characteristics of the nanoemulsion or not. The *p*-value is used to determine statistical significance, and the analysis results will help select an appropriate model and interpret the significance of factors that influence the characteristics of nanoemulsions, which can be seen in the table.

Based on the ANOVA RSM analysis of three factors, namely the type of Tween in nanoemulsion, Tween concentration, and Parijoto extract concentration, all ANOVA values show probabilities <0.0001 (*p* < 0.05). This indicates that the quadratic response surface model used for both responses (dependent variables) is significant and can be used to optimize extraction factors (32). The coefficient of determination, or R square, depicts how independent data can explain dependent data. The range of R square values is between 0 and 1, where values closer to 1 indicate better explanatory power.

In the Central Composite Design analysis, the p-value indicates the significance of each coefficient in the built polynomial regression model. The lower the p-value, the more significant the contribution of the coefficient to the overall regression model (33). Using experimental data within the allowed range of variables in this study to create mathematical equations, which may have broader general applications, can provide the ability to predict system behavior when different factors are combined. From the perspective of optimizing the formation of emulsion nanoparticles, there is potential to develop more significant results based on the variables investigated in this study. Additionally, this optimization may be performed using the techniques outlined in this research to test further the effects of time and temperature or other conditions, as needed.

Table 2 shows details of the RSM approach used to assess particle size (nm), Poly Dispersity Index, Ζ-potential (mv), Conductivity, pH, and viscosity (Cp) in nanoemulsion of Parijoto fruit extract involved in a series of 81 experiments based on factorial design. The coefficients for the second-degree polynomial Equation are determined through experimental results, along with the regression coefficients for Particle Size (Y1), Poly Dispersity Index (Y2), Ζ-potential (Y3), Conductivity (Y4), pH (Y5), and viscosity (Y6). The Equation presented as Equation (2) shows the full quadratic model, while Table 2 shows the models predicting the response of the independent variables (Y1–Y6).

**Table 2:** ANOVA (analysis of variance) for the RSM quadratic model particle size, poly dispersity index, Ζ-potential, conductivity, pH, and viscosity in nanoemulsion.

Quadratic model equation	Sources of variation	*p*-value
Particle size (*R*^2^: 0.558 *R*^2^_a_: 0.50156) Y_1_ = −0.000008–0.000069X_1_ + 0.000040X_2_+ 0.000032X_3_ + 0.000056X_1_^2^+ 0.000064X_2_^2^–0.000003×_3_^2^–0.000056X_1_X_2−_0.000044X_2_X_3_ + 0.000065X_2_X_3_	Model	0,294*
	*Lack of fit*	0,185
*Poly dispersity index* (*R*^2^: 0.3643 *R*^2^_a_: 0.2471) Y_2_ = 6.23086 + 0.58801 X_1_–0.75655 X_2_+ 84.3654 X_3_ + 24.65 X_1_^2^+ 18.7663 X _2_^2^–20.744 X_3_^2^ + 23.0025 X_1_X_2_ + 26.3043 X_2_X_3_ + 9.5269 X_2_X_3_	Model	0,041*
	*Lack of fit*	0,692
*Ζ-potential* (*R*^2^: 0.54003 *R*^2^_a_: 0.56905) Y_3_ = 0.000062–0.000023 X_1_–0.000010 X_2_+ 0.000008 X_3_ + −0.000007 X_1_^2^+ 0.000003 X_2_^2^ + 0.000008 X_3_^2^ + −0.000006 X_1_×_2−_0.000008 X_2_X_3_ + −0.000005 X_2_X_3_	Model	0,000*
	*Lack of fit*	0,980
*Conductivity* (*R*^2^: 0.2444 *R*^2^_a_: 0.3464) Y_4_ = 4035.80–1198.06X_1_ + 833.22X_2_–1083.49X_3_–2597.39X_1_^2^–709.42X_2_^2^ + 881.10X_3_^2^ + 305.68X_1_X_2−_700.69X_2_X_3_–943.96X_2_X_3_	Model	0,0004*
	*Lack of fit*	0,928
pH (*R*^2^: 0.832 *R*^2^_a_: 0.797) Y_5_ = 0.003122 - 0.000040X_1_–0.000060X_2_+ 0.000039×_3_–0.000034×_1_^2^+ 0.000047X_2_^2^ + 0000031 X _3_^2^–0.000006X_1_X_2−_0.000015X_2_X_3_ + 0000031- X_2_X_3_	Model	0,000*
	*Lack of fit*	0,067
Viskositas (*R*^2^: 0.95976 *R*^2^_a_: 0.95466) Y_6_ = 0.015177 – 0.009573X_1_–0.003288X_2_–0.000624X_3_–0.008334X_1_^2^–0.000266X_2_^2^–20.744 X_3_^2^ + 23.0025 X_1_X_2_ + 26.3043 X_2_X_3_ + 9.5269 X_2_X_3_	Model	0,000*
	*Lack of fit*	0,103

*The model has a statistically significant effect (*p* ≤ 0.05). **Model mismatch or lack of fit occurs (*p* ≤ 0.05).

To assess the extent to which the equation model in RSM fits the data and how strong the influence of the variables is, the coefficient of determination or (R2) is used. Chin (34) has categorized that for model suitability, the R-Square value is substantial if it is more than 0.67, moderate if it is more than 0.33 but lower than 0.67, and weak if it is more than 0.19 but lower than 0.33. pH and viscosity indicate strong model adequacy on these response variables. In contrast, the responses of Particle Size, Poly Dispersity Index, Ζ-potential, and Conductivity indicate a moderate model for these response variables. A lack of fit test was then performed to assess model fit for each response. With a *p*-value exceeding 0.05, it was confirmed that the model adequately fit the experimental data, as seen in Table 2.

### 3.3 Contour plot on particle size, poly-dispersity index, Ζ-potential, conductivity, pH, and viscosity as a function of nanoemulsion parijoto fruit extract

**Figure 1:**
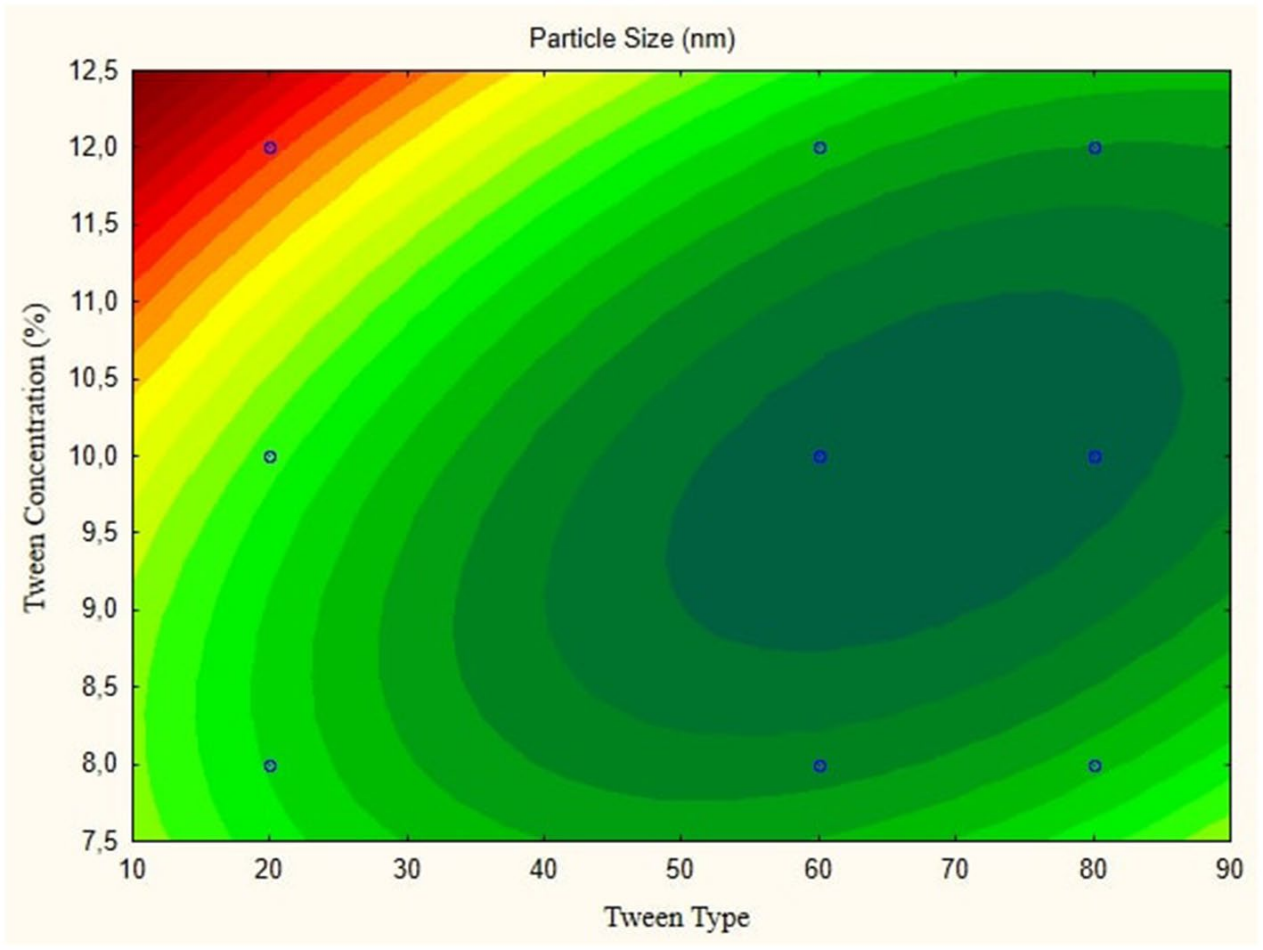
Contour plot of particle size is a function of nanoemulsion.

**Figure 2:**
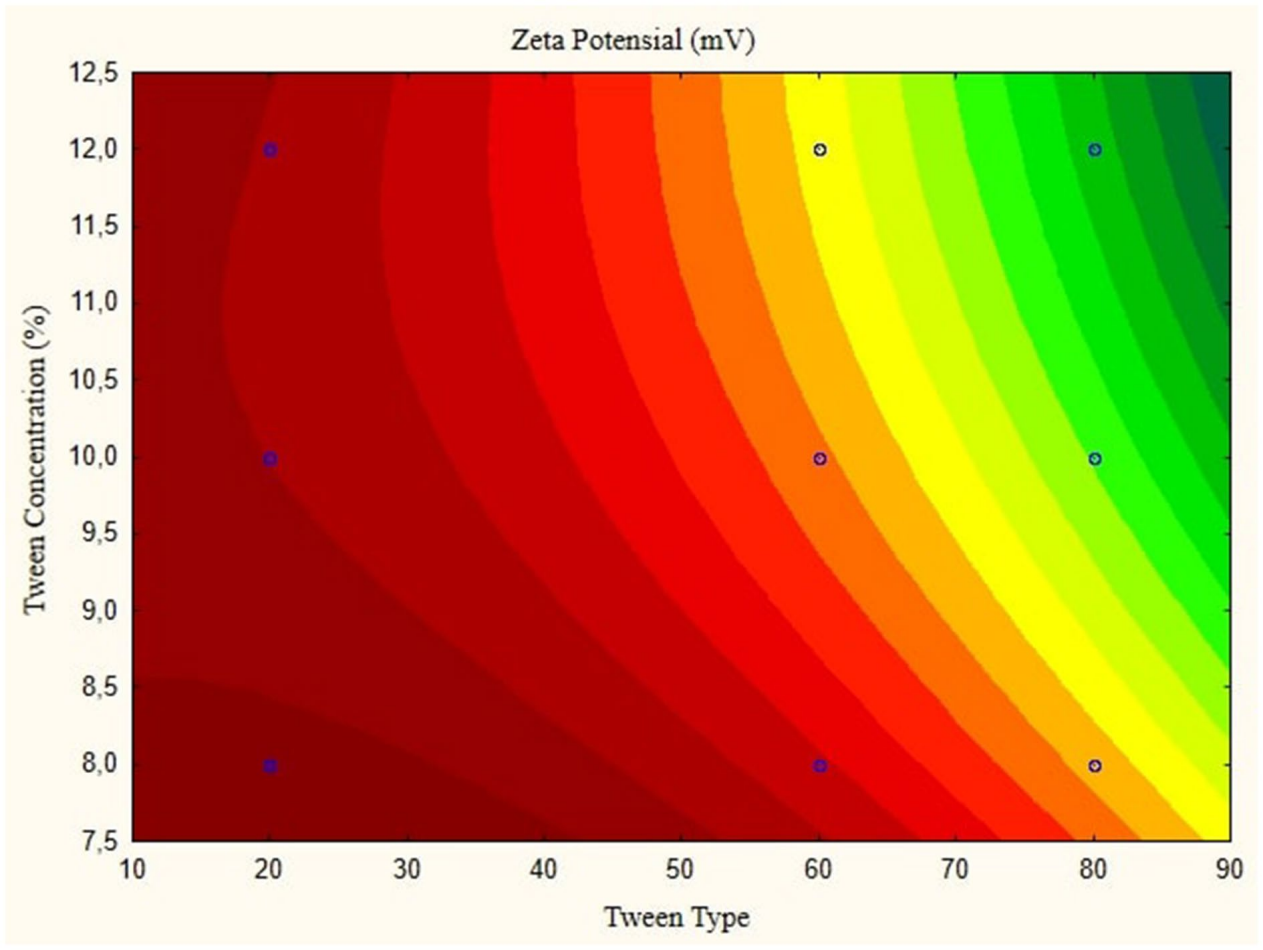
Contour plot of zeta potential as a function of nanoemulsion parijioto fruit extract.

**Figure 3:**
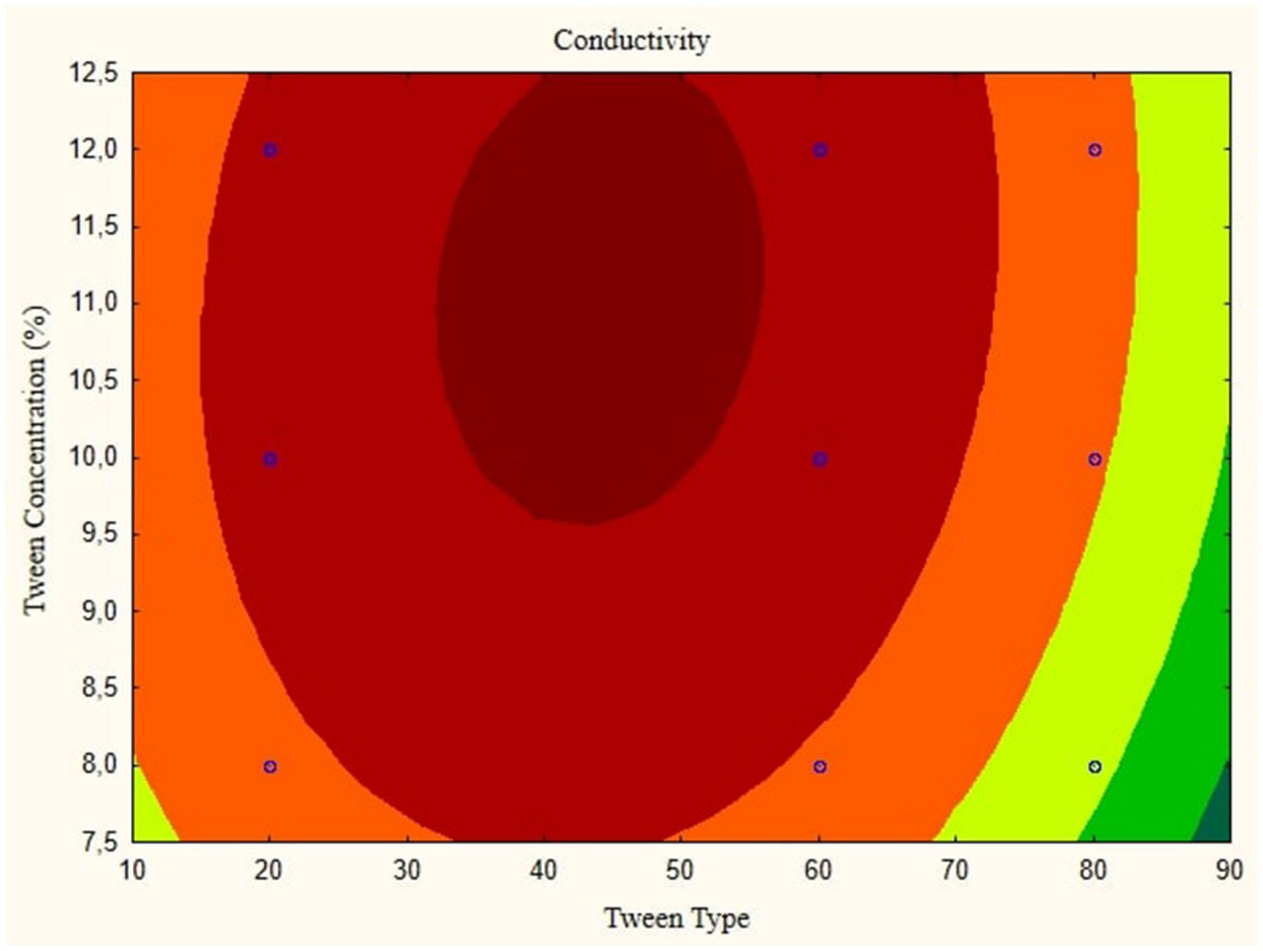
Contour plot of conductivity as a function of nanoemulsion parijioto fruit extract.

**Figure 4:**
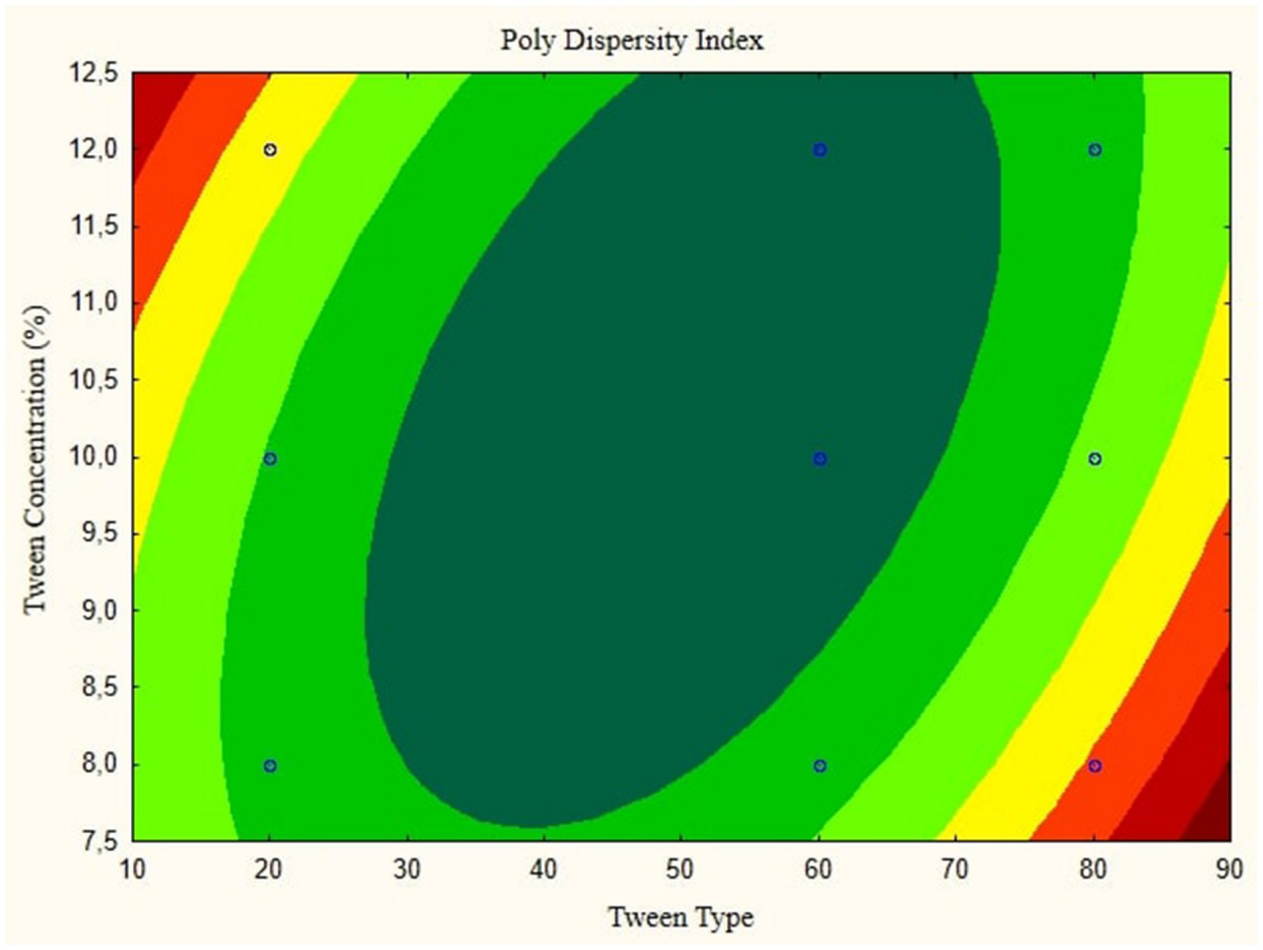
Contour plot of poly dispersity index as a function of nanoemulsion of parijioto fruit extract.

**Figure 5:**
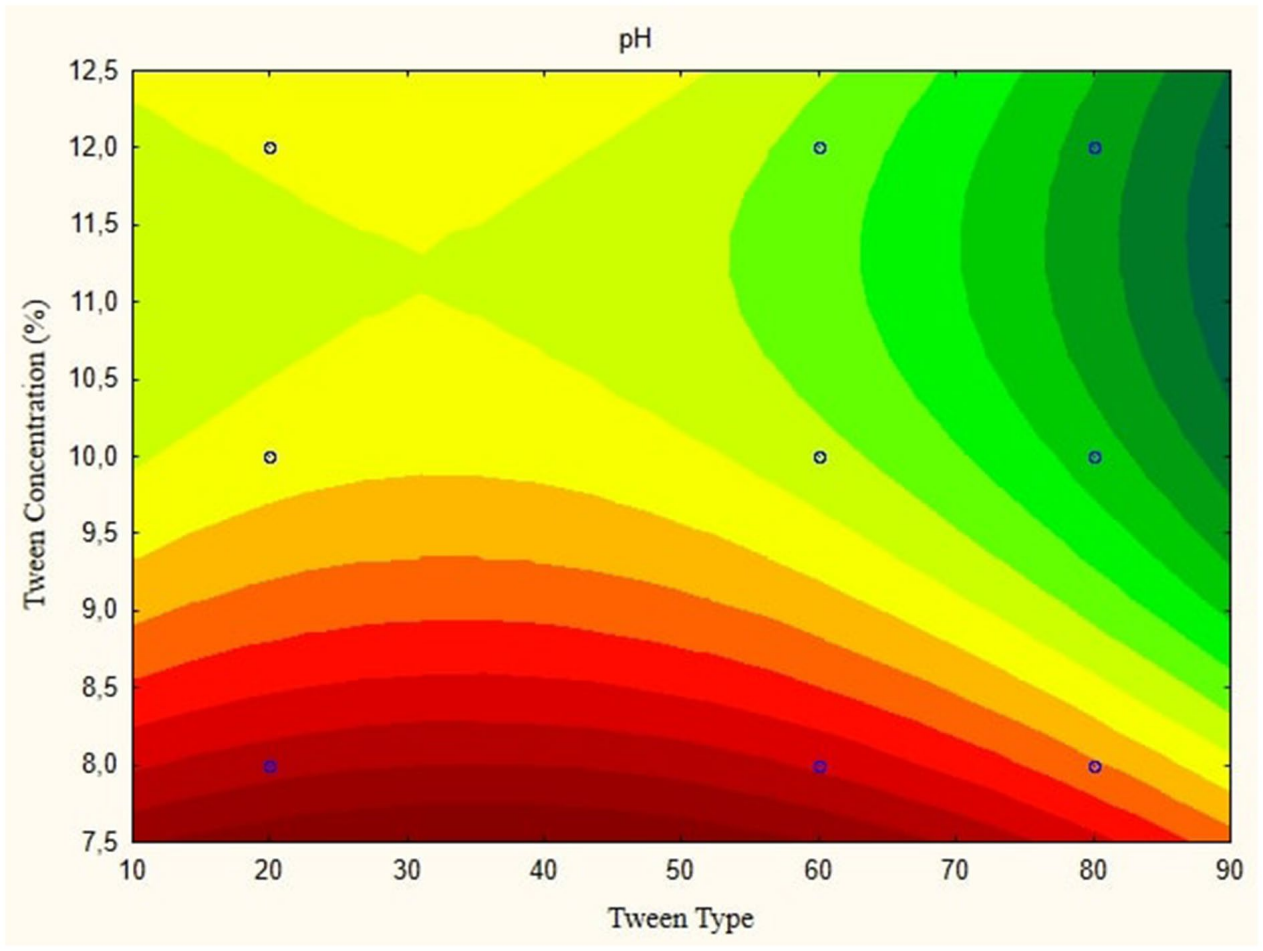
Contour plot of pH function in nanoemulsion of parijioto fruit extract.

**Figure 6:**
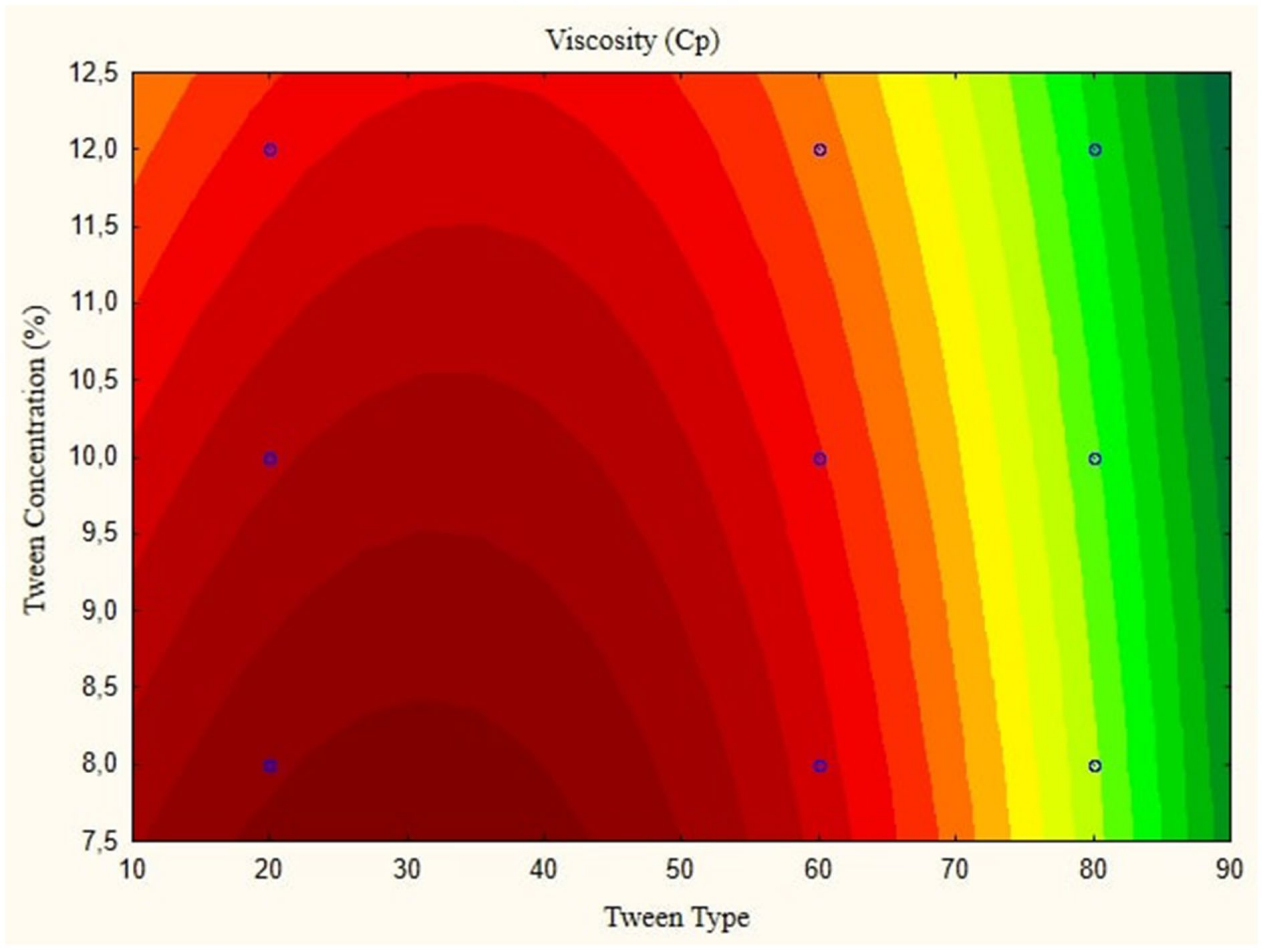
Contour plot viscosity function in nanoemulsion parijioto fruit extract.

In this research, the model is created as a Contour plot, showing the response: Particle Size, Poly Dispersity Index, Ζ-potential, Conductivity, pH, and Viscosity. Continued research shows a significant relationship between particle size and tween concentration and the type of lipophilic Tween in nanoemulsions, as shown in Figures 1–6. The presented data offers valuable insights into the influence of lipophilic tween type and tween concentration on various properties of the nanoemulsion derived from parijoto fruit extract. Each figure depicts the contour plots illustrating the interaction effects between these two factors on different characteristics of the nanoemulsion.

Figure 1, the contour plot demonstrates the interaction between the lipophilic tween type and tween concentration in controlling nanoparticle size. It reveals that as the lipophilic tween type increases from 20 to 80, and the tween concentration rises from 8 to 10%, there is a general trend of increasing particle size, albeit with a slight decreasing trend observed to some extent. This suggests that both factors play a role in determining the nanoparticle size, with higher concentrations leading to larger particle sizes. Moving to Figure 2, which illustrates the Ζ-potential of the nanoemulsion, an increase in the lipophilic Tween type from 60 to 80 and an increase in tween concentration from 8 to 10% correspond to an increase in Ζ-potential. Interestingly, no further changes are observed beyond this point. This indicates that these specific conditions result in optimal Ζ-potential, possibly indicating enhanced stability of the nanoemulsion.

Figure 3 showcases the influence of lipophilic tween type and tween concentration on the conductivity of the nanoemulsion. As the lipophilic tween type increases from 20 to 80 and the tween concentration rises from 8 to 12%, conductivity is consistent without any further changes. This suggests a direct relationship between these factors and the conductivity of the nanoemulsion. The Contour plot presented in Figure 4 demonstrates the effect of lipophilic tween type and tween concentration on the nanoemulsion’s Poly Dispersity Index (PDI). Interestingly, an increase in lipophilic tween type from 60 to 80 and a decrease in tween concentration from 12 to 8% lead to an increase in PDI value without further changes. This indicates a complex interaction between these factors in determining the homogeneity of particle size distribution within the nanoemulsion.

Figure 5 depicts the pH contour plot of the parijoto fruit extract nanoemulsion. An increase in lipophilic Tween type from 20 to 80 and an increase in tween concentration from 8 to 12% result in a consistent increase in pH without any further changes. This observation suggests that these specific conditions contribute to the alkalinity of the nanoemulsion, which may have implications for its stability and functionality. Finally, Figure 6 illustrates the viscosity contour plot of the nanoemulsion. An increase in lipophilic tween type from 35 to 80 and an increase in tween concentration from 8 to 12% lead to an increase in viscosity without further changes. This indicates that higher concentrations of lipophilic Tween and Tween result in a thicker consistency of the nanoemulsion, which affects its flow properties and application. The presented data highlights the intricate relationship between lipophilic tween type and tween concentration in influencing various physicochemical properties of the nanoemulsion derived from parijoto fruit extract. These findings provide valuable insights for optimizing the formulation and manufacturing process of the nanoemulsion for potential applications in various industries.

Research on the influence of surfactant type and concentration on nanoemulsion indicates that the selection of surfactant significantly affects the characteristics of nanoemulsion. Various surfactant types, such as Tween 20, Tween 60, and Tween 80, play different roles in forming nanoemulsions. The research results show that the particle size of Tween 80 surfactant is the highest, with an average particle size of 107.196 nm. Similar results were reported by Chang et al. (35), who obtained the smallest droplets in carvacrol-based nanoemulsion made with a mixture of food-grade non-ionic surfactants (Tween 20, 40, 60, 80, and 85). Tween, a non-ionic surfactant derived from sorbitan ester, is soluble or dispersible in water and is commonly used as an oil-in-water emulsifier in the pharmaceutical, cosmetic, and cleaning industries. Among these surfactants, Tween 80 is one of the most commonly used. Research by Jadhav et al. (36) confirms that the type of non-ionic surfactant significantly influences the average particle diameter of the formed colloid dispersion. The smallest droplets were observed in systems prepared using Tween 80, while the largest droplets formed in systems using Tween 85. The surfactant’s Hydrophilic–Lipophilic Balance (HLB) plays a role in forming small particles. Surfactants with either too high (Tween 20) or too low (Tween 85) HLB values cannot form optimal nanoemulsions. Tween types with intermediate HLB values (40, 60, and 80) can form nanoemulsions with small particle sizes. However, there is no strong correlation between HLB values and particle sizes produced by these surfactants. Small-molecule surfactants have higher surface activity and form smaller emulsion droplets than large ones (37).

Another critical factor for minimal droplet emulsion formation is the Hydrophilic–Lipophilic Balance (HLB) value of the surfactant, defined by Griffin as the ratio of surfactant hydrophilicity to lipophilicity. A high HLB value indicates strong hydrophilicity, and the HLB values of non-ionic surfactants generally range from 0 to 20, such as Tween 20 (HLB 16.7) and Tween 80 (HLB 15) (38). Two polymer and particle surface tension mechanisms influence emulsion stability: steric stability caused by macromolecules adsorbed on particle surfaces and electrostatic stability due to repulsion between surface-charged droplets. In nanoemulsions made with Tween 80 surfactant, the surfactant may not have a charge on the hydrophobic group, causing the covered droplet surface to be non-charged and resulting in low ζ-potential values, which can lead to increased particle size and PDI.

However, a different study proposed by Alam et al. (39) suggests that Tween 20 helps improve PDI and allows for minimum polydispersity. Compared to other nanoparticles, the ability to maintain particle integrity using Tween 20 is significant. Increasing the Surfactant content in the formulation increases the polydispersity indices for natural extracts in the 3D response surface graph. This indicates that the use of Tween types with low and high HLB values can be applicable when combined with an optimal concentration of co-surfactant.

Surfactant concentration is also a critical factor in nanoemulsion formation. Research indicates that increasing surfactant concentration can result in smaller and more homogenous size distribution. However, there is a specific limit where surfactant concentration reaches a plateau level, leading to unadsorbed surfactant aggregation and micelle formation. The results show that the higher the Tween concentration, the higher the size and PDI. This is confirmed by Abaolnaja et al. (40), stating that nanoemulsions prepared with higher surfactant concentrations significantly increase short-term stability. Systems with 15 or 20% weight of Tween 80 are highly unstable to increasing dilution, indicating that a medium surfactant concentration level may be more suitable for stable nanoemulsion preparation. Although the initial droplet size is small, higher surfactant concentrations can increase raw material costs and cause undesirable sensory (taste) issues in commercial applications. Therefore, this study uses a 10% weight of Tween 80 in further experiments.

Increasing surfactant concentration increases the number of surfactant molecules migrating from the oil phase to the emulsion water phase, and nanodroplets form. Frictional forces applied to the oil–water interface, coated with emulsifier, cause some emulsifiers to sink parallel to the surface layer while others detach from the surface layer. Hasani et al. reported that droplet size increases by increasing surfactant concentration to 20%, and particles have a broad and non-uniform size distribution. The instability of nanoemulsion at high surfactant concentrations may be related to the depletion-flocculation mechanism of absorbed surfactant. With increased surfactant concentration, additional surfactant molecules form micelles in the continuous phase rather than orienting on the particle surface. This leads to an increase in local osmotic pressure, causing the continuous phase between moving droplets to decrease, reducing the continuous phase between those droplets. As a result, aggregation occurs, causing an increase in particle size. According to Oh et al. (41) and Tadros et al. (42), the average droplet size becomes smaller, and the size distribution becomes narrower with increasing emulsifier concentration, ultimately reaching a plateau level. Beyond the plateau level, free or unadsorbed emulsifiers may accumulate to form micelles. Nanoemulsions are known to be thermodynamically unstable, tending to minimize interfacial area through coalescence.

An increase in the filler extract’s concentration can lead to nanoparticles’ tendency to aggregate or form agglomerates and pH nanoemulsion. This phenomenon may occur due to physical or chemical interactions between nanoparticles and compounds in the filler extract. Increase in extract concentration results in an increase in particle size, particularly at the highest concentration of 347.2 nm. On the other hand, the smallest concentration has the lowest particle size at 86.98 nm. These results indicate that higher concentrations may increase the likelihood of particle agglomeration.

Furthermore, increasing the concentration of parijoto fruit extract can increase the total mass in the solution, which, in turn, can increase overall viscosity. Additional particles or molecules from the filler extract can contribute to the increase in viscosity. Particles with the highest concentration have the highest viscosity and vice versa. This increase in viscosity may be caused by excess extract loaded into particles. The physicochemical characteristics of the filler extract may influence the viscosity properties of nanoparticles, and factors such as changes in pH, temperature, or chemical composition may also play a role in viscosity increase. Parijoto fruit is rich in active compounds, such as anthocyanins, which can affect the surface charge of nanoemulsion particles. At a certain pH, anthocyanins or other components may have specific charges that can influence the electrostatic stability of particles (43). Anthocyanins may undergo solubility changes at specific pH values, affecting the distribution and stability of the nanoemulsion’s oil or water phase. The same occurs with surfactants, where variations in charge of the filler extract from parijoto fruit can affect the interaction between nanoparticles, anthocyanins, and other components in the system. The loading capacity of the extract in the nanoemulsion likely depends on its solubility in the system used. Anthocyanins tend to undergo color changes with pH (pH-dependent color shift). Additionally, the antioxidant activity of anthocyanins can be influenced by pH. This complexity can modulate the overall physicochemical properties of the nanoemulsion system.

Nanoemulsions, despite their promising applications, present challenges related to stability. The challenge is the propensity for Ostwald ripening, wherein larger droplets grow at the expense of smaller ones, leading to phase separation and reduced shelf-life. Additionally, factors such as temperature fluctuations, pH changes, and exposure to light can exacerbate instability, causing particle aggregation. Surfactant degradation over time is another concern, as it can compromise the emulsion’s ability to maintain a stable dispersion. However, the industrial application of parijoto fruit or extract holds significant potential. Parijoto fruit, known for its rich content of bioactive compounds, including anthocyanins, flavonoids, and phenolic acids, offers various health benefits such as antioxidant and anti-inflammatory properties. Incorporating parijoto extract into nanoemulsions can enhance its bioavailability and efficacy, making it suitable for a range of industrial applications especially food functional and nutracetical.

### 3.4 Optimal point prediction from RSM in nanoemulsion parijoto fruit extract

**Figure 7:**
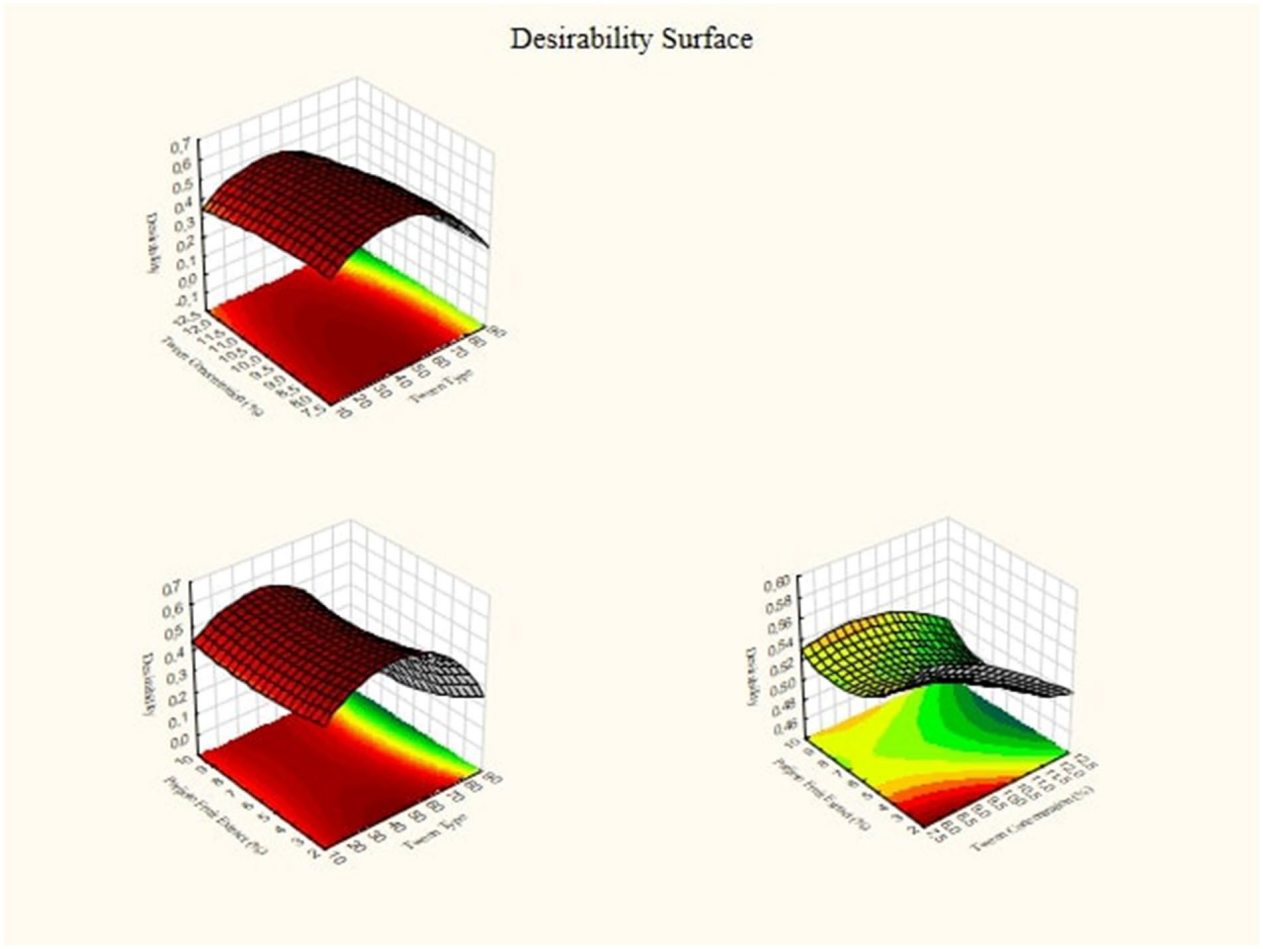
3D desirability profile and response graphs for nanoemulsion parijioto fruit extract.

Optimal point predictions from the Response Surface Methodology are obtained by combining optimal conditions based on interactions between independent variables. Profiler predictions are obtained if the fitted surface graph is in minimum, maximum, or saddle form. 3D graphics on Figure 7 shows a complex interaction between the variable factors of lipophilic tween type and tween concentration on the response. Increasing the lipophilic tween type value increases the response somewhat, but the tween concentration value can modify the effect. There is an optimal region where the response reaches its peak. The implication for practice is that by setting the variable factors at levels that are estimated to be optimum, the research results can achieve the highest optimization in the desired response, which can be seen in Figure 7.

It can be seen in Table 3 that to achieve the maximum desired concentration of nanoparticle size, ζ-potential, Conductivity, Poly Dispersity Index, degree of acidity, and Viscosity, it is necessary to set the Tween solvent concentration to 80, Tween concentration to 12% and Parijoto fruit extract concentration to 7. 5%. This set of conditions has a desirability value of 0.74. Because the value is almost close to 1 and falls into the moderate category, this set of conditions is quite optimal for the aim of this research, namely to maximize the response.

**Table 3:** Prediction of optimum conditions for parijoto fruit extract nanoemulsion.

Types of analysis	Types of lyphophilic tweens	Tween concentration (%)	Parijoto fruit extract concentration (%)	Nanoparticle size (nm)	Ζ-potential (mV)	Conductivity (mS/cm)	Poly dispersity index	Degree of acidity (pH)	Viscosity (Cp)	Desirability value
Optimum condition prediction	80	12	7.5	61.97	−28.48	0.082	0.691	6.864	5.668	0.74
Maximum value at optimum conditions	80	12	7.5	39.94	−32.48	0.048	0.371	6.82	5.422	
Minimum value at optimum conditions	80	12	7.5	163.88	−26.37	0.115	1.011	6.9	5.913	

The optimization of nanoemulsion formation from Parijoto fruit extract using Response Surface Methodology (RSM) has been conducted in this study. RSM is a statistical method used to design experiments and analyze the impact of multiple independent variables on a measured response. As an output of this research, the synthesis process conditions of nanoemulsion from Parijoto fruit extract can be optimized to achieve particle size, polydispersity index (PDI), ζ-potential, conductivity, pH, and viscosity levels. RSM determines the optimal extraction time and temperature to maximize the response variable outcomes (44). In line with this, predictions and observations are within a narrow range and do not show significant differences at a 5% significance level, indicating the model’s suitability for optimization and process efficiency purposes.

The optimal point prediction from the Response Surface Methodology is obtained by integrating optimal conditions and depends on the interaction between independent variables, as Ratnawati et al. (45) explained. The prediction profile is formed when the adjusted surface graphs show a minimum, maximum, or saddle shape. The optimization process can achieve optimal responses by analyzing each response beforehand, ultimately reducing effort and operational costs, as Nurmiah et al. (46) stated. Desirability, with a range of values from 0 to 1, is used as the optimization target value, with low (0–0.49), moderate (0.5–0.79), and high (0.8–1) categories. The closer the value of 1 is, the greater the desirability, which indicates the suitability of the combination of process parameters to achieve optimal response variables.

Table 3 shows that to achieve the desired concentrations of nanoparticle size, ζ-potential, conductivity, polydispersity index, acidity level, and viscosity, Tween 80 with a Tween concentration of 12% and Parijoto fruit extract concentration of 7.5% is necessary. This set of conditions has a desirability value of 0.740349. Since its value is close to 1 and falls into the moderate category, this set of conditions is optimal for this research to maximize the response.

## 4 Conclusion

In this series of experiments, nanoemulsion from parijoto fruit has been characterized, considering various physicochemical parameters such as particle size, polydispersity index, ζ-potential, conductivity, pH, and viscosity, respectively, ranged from 14,603 ± 16.73 nm to 118,053 ± 4.5825 nm, 0.402 ± 0.038 to 0.874 ± 0.100, −22.197 ± 0.738 mV to −28.207 ± 1.598 mV, 0.064 ± 0.013 to 0.090 ± 0.010 mS/cm, and 6.747 ± 0.035 to 6.897 ± 0.006, and 3.827 ± 0.021 to 5.633 ± 0.058. The research results indicate significant variations in the physical characteristics of both nanomaterials regarding changes in surfactant and parijoto extract concentrations. Increased surfactant concentration tends to produce smaller particle sizes and a more homogeneous distribution, although certain limitations were found that lead to surfactant aggregation and micelle formation. The nanoemulsion characteristics include ζ-potential, polydispersity, particle size, conductivity, pH, and viscosity. The type and concentration of surfactants played a crucial role in determining the properties of the nanoemulsions. Variations in surfactant parameters resulted in observable differences in emulsion characteristics, highlighting the importance of surfactant selection and optimization. To achieve optimal nanoemulsion process conditions, it is recommended to use Tween 80 with 12% Tween concentration and 7.5% parijoto fruit extract concentration, resulting in a desirability value of 0.74, into the moderate category.

## Data availability statement

The datasets presented in this article are not readily available because non-commercial use: the dataset is provided solely for academic research purposes. Requests to access the datasets should be directed to kristina@unika.ac.id.

## Author contributions

VA: Conceptualization, Funding acquisition, Investigation, Methodology, Project administration, Resources, Supervision, Validation, Writing – original draft, Writing – review & editing. AP: Data curation, Formal analysis, Methodology, Project administration, Validation, Writing – original draft, Writing – review & editing. BS: Data curation, Formal analysis, Methodology, Validation, Writing – original draft, Writing – review & editing. YP: Data curation, Formal analysis, Investigation, Resources, Software, Validation, Visualization, Writing – original draft, Writing – review & editing.
